# Regulation of CD4 Receptor and HIV-1 Entry by MicroRNAs-221 and -222 during Differentiation of THP-1 Cells

**DOI:** 10.3390/v10010013

**Published:** 2017-12-30

**Authors:** Robert Lodge, Julian C. Gilmore, Jérémy A. Ferreira Barbosa, Félix Lombard-Vadnais, Éric A. Cohen

**Affiliations:** 1 Institut de recherches cliniques de Montréal, Montreal, QC H2W 1R7, Canada; robert.lodge@ircm.qc.ca (R.L.); julian.gilmore@ircm.qc.ca (J.C.G.); jeremy.ferreirabarbosa@ircm.qc.ca (J.A.F.B.); felix.lombard-vadnais@mail.mcgill.ca (F.L.-V.); 2 Department of Microbiology, Infectiology and Immunology, Université de Montréal, Montreal, QC H3T 1J4, Canada

**Keywords:** microRNA, miR-221, miR-222, THP-1 cell line, macrophage, monocyte, cluster of differentiation 4 (CD4), RNAseq, human immunodeficiency virus type-1 (HIV-1)

## Abstract

Human immunodeficiency virus type-1 (HIV-1) infection of monocyte/macrophages is modulated by the levels of entry receptors cluster of differentiation 4 (CD4) and C-C chemokine receptor type 5 (CCR5), as well as by host antiviral restriction factors, which mediate several post-entry blocks. We recently identified two microRNAs, miR-221 and miR-222, which limit HIV-1 entry during infection of monocyte-derived macrophages (MDMs) by down-regulating CD4 expression. Interestingly, CD4 is also down-regulated during the differentiation of monocytes into macrophages. In this study, we compared microRNA expression profiles in primary monocytes and macrophages by RNAseq and found that miR-221/miR-222 are enhanced in macrophages. We took advantage of the monocytic THP-1 cell line that, once differentiated, is poorly susceptible to HIV-1. Accordingly, we found that CD4 levels are very low in THP-1 differentiated cells and that this down-regulation of the virus receptor is the result of miR-221/miR-222 up-regulation during differentiation. We thus established a THP-1 cell line stably expressing a modified CD4 (THP-1-CD4^R^) that is not modulated by miR-221/miR-222. We show that in contrast to parental THP-1, this line is productively infected by HIV-1 following differentiation, sustaining efficient HIV-1 CD4-dependent replication and spread. This new THP-1-CD4^R^ cell line represents a useful tool for the study of HIV-1-macrophage interactions particularly in contexts where spreading of viral infection is necessary.

## 1. Introduction

Macrophages make up a heterogeneous population of cells and are important components of innate immunity. Although macrophages are targets of human immunodeficiency virus type-1 (HIV-1) infection, infected macrophages are more resistant to virus-mediated cytopathic effects and are not significantly depleted in vivo [1,2]. As such, they are thought to promote viral spread and the establishment of persistent viral reservoirs [3].

The stage of differentiation as well as the activation status of macrophages profoundly affect their susceptibility to HIV-1 infection [3]. While cells of the monocyte lineage in peripheral blood are rarely infected in vivo [1,4], differentiation of monocytes into macrophages in some tissues, such as the brain or lung, can result in susceptibility to infection [5]. Monocytes in culture must also undergo differentiation into macrophages to become maximally susceptible to productive infection [6,7]. While the HIV-1 receptor cluster of differentiation 4 (CD4) and virus co-receptors C-C chemokine receptor type 5 (CCR5) and C-X-C chemokine receptor type 4 (CXCR4) are expressed on monocytes and macrophages [8,9], their expression levels are substantially modulated during differentiation. Hence, CXCR4 and CD4, are relatively abundant on monocytes but their expression levels are reduced during differentiation into macrophages [7,9]. In contrast, CCR5, which is expressed at lower levels on fresh monocytes is up-regulated in monocyte-derived macrophages [7,8,9]. Although CCR5 acts as the principal co-receptor during HIV-1 infection of macrophages, its usage is not sufficient to predict macrophage tropism (M-tropic viruses) [10,11]. Indeed, macrophages express 20-fold less CD4 as opposed to T lymphocytes, requiring M-tropic viruses to encode Env glycoproteins with increased affinity for CD4 or enhanced exposure to CCR5 following CD4 engagement [12,13,14]. Thus, the low levels of CD4 at the surface of macrophages appears to limit the ability of most CCR5-tropic HIV-1 to mediate entry into these cells.

Although monocytes generally express higher levels of CD4 than monocyte-derived macrophages (MDMs) [15,16], their resistance to HIV-1 replication in vitro is primarily due to expression of antiviral restriction factors, affecting viral replication at or before reverse transcription. Indeed, monocytes express high levels of restriction factors, particularly apolipoprotein B mRNA editing enzyme, catalytic polypeptide-like, type 3A (APOBEC3A), which is down-regulated upon monocyte differentiation into MDMs [17,18]. Similarly, expression of antiviral host factors such as sterile alpha-motif, histidine-aspartic domain-containing protein 1 (SAMHD1) that reduces the pool of cellular deoxyribonucleotides available for reverse transcription influence the levels of productive HIV-1 replication in macrophages [19,20]. In addition to such restriction factors, recent studies have also implicated naturally occurring cellular microRNAs (miR) in modulating the susceptibility of monocyte and macrophages to HIV-1 infection [21,22,23,24].

Recently, we and others reported that two microRNAs, miR-221 and miR-222, were targeting the 3′ untranslated region (UTR) of CD4 mRNAs and as a result induced a down-regulation of CD4 [25,26]. MiR-221 and miR-222 are processed from a single pri-microRNA precursor and contain the same mRNA-targeting seed sequence [27,28]. Furthermore, these two small RNAs are enhanced by NF (nuclear factor)-κB activating agents, such as tumor necrosis factor (TNF)-α [26,27,29,30]. Hence, the release of TNF-α in HIV-1 infected MDM cultures enhances miR-221/miR-222 expression in HIV-1 non-producing bystander cells and limits HIV-1 replication and spread [26].

In the present study, we investigated the mechanism governing the reduction of CD4 expression during monocyte to macrophage differentiation. We show that miR-221 and miR-222 are among the up-regulated microRNAs following monocyte differentiation into macrophages, both in primary cells and in the monocytic THP-1 model cell line. In THP-1 cells, CD4 expression is strongly down-regulated during differentiation primarily by miR-221/miR-222. THP-1-CD4^R^ cells that express a *CD4* transgene independent of miR-221/miR-222 modulation, maintain high levels of CD4. Such differentiated THP-1-CD4^R^ cells sustain efficient productive infection by CCR5-tropic HIV-1 following differentiation as opposed to differentiated THP-1 cells, which are poorly permissive to infection by these viruses. The development of the THP-1-CD4^R^ cell line will advance the study of HIV-1-macrophage interactions particularly in contexts where spreading of viral infection is necessary.

## 2. Materials and Methods

### 2.1. Plasmid Constructs, MicroRNA Mimics and Antagomirs

The NL4-3 [31], NL4-3Env-Luc+Vpr+ [32], SVCMV-VSV-G [33], SVIII-ADA-Env [34], NL4-3-IRES-GFP and NL4-3ADA-IRES-GFP [35,36] constructs were previously described. The Vpr-negative version of NL4-3ADA-IRES-GFP encodes a truncated 27-amino acid long Vpr [37].

The miRCURY locked nucleic acid (LNA) inhibitors for miR-221-3p (#4103821-002) and miR-222-3p (#4101984-002) (antagomirs) were purchased from Exiqon (Vedbaek, Denmark). Non-targeting control RNAs were obtained from Dharmacon/GE Healthcare (siGENOME non-targeting #2, D-001210-02, Lafayette, CO, USA) or Ambion/ThermoFisher Scientific (#AM16104, Waltham, MA, USA).

### 2.2. Antibodies and Chemicals

The following antibodies were used in flow cytometry assays: mouse anti-human CCR5, anti-human CXCR4 or anti-human CD4 (OKT4) (all PerCP-Cy5.5), mouse anti-human CD11c-PE-Cy7, mouse anti-human CD14-Pacific Blue and mouse anti-human CD16-APC-Cy7, all with corresponding isotype controls (Biolegend, San Diego, CA, USA). Fixable AquaDead or YellowDead Stain kits were from Molecular Probes (Invitrogen/Life Technologies, Waltham, MA, USA).

### 2.3. Virus Production

Human embryonic kidney (HEK) 293T cells were maintained in Dulbecco’s modified Eagle medium (DMEM, Wisent, St-Bruno, QC, Canada) supplemented with 10% fetal bovine serum (FBS, Wisent) at 37 °C in a 5% CO_2_ incubator. Viruses were obtained by calcium phosphate transfection of relevant proviral constructs and virus-containing supernatants recovered following 60 h of transient expression. Supernatants were cleared of cells, filtered and virus pellets obtained following ultracentrifugation on a 20% sucrose cushion. Viruses were re-suspended in phosphate buffered solution (PBS) and aliquots kept at −80 °C. Virus titers were determined using the TZMbl reporter cell line [38] either by β-galactosidase expression or green fluorescent protein (GFP) expression in the case of GFP-expressing viruses.

### 2.4. Establishment of a THP-1-CD4^R^ Cell Line Expressing CD4 that Is Resistant to MiR-221/222 Modulation

Multistep polymerase chain reaction (PCR) was performed on a plasmid containing the CD4 coding sequence in order to obtain a cDNA lacking *CD4* 5′ untranslated region (UTR) but encompassing a gene encoding a carboxy-terminally V5-tagged CD4, flanked by the BamHI and EcoRI restriction sites (see Table 1 below). This cDNA was inserted into the pENTR1A shuttle vector and transferred by recombination into the lentiviral vector pLentiCMV/TO Hygro DEST2 in order to generate pLentiCD4-V5. Lentiviruses were produced by triple transfection of pLentiCD4-V5, psPAX2 and SVCMV-VSV-G in HEK293T cells as described previously [39]. Control lentiviruses were also produced using the empty lentiviral vector instead of pLentiCD4-V5. Thereafter, lentiviral p24 was quantified and THP-1 cells were transduced by spinoculation (1200 g for 2 h at room temperature with 8 μg/mL polybrene) with 1 μg of either control or CD4-V5-expressing lentiviral vectors. THP-1-Control (THP-1^ve^) or THP-1-CD4^R^ cells were obtained following hygromycin (500 μg/mL) selection and maintained in Roswell Park Memorial Institute 1640 medium (RPMI-1640, Wisent) supplemented with 10% FBS and hygromycin (50 μg/mL). Differentiation of THP-1 and THP-1-CD4^R^ was obtained by treating cells with 50 nM phorbol 12-myristate 13-acetate (PMA) for 24 h and further incubating cells without PMA for an additional 48 h prior to use.

### 2.5. Monocytes, CD4+ T Cells and MDMs Isolation; MDM and THP-1 Transfection and HIV-1 Infection

Peripheral blood samples and blood that was leukocyte-enriched by leukapheresis were obtained from HIV-1- and hepatitis C virus-seronegative adults who had given written informed consent. Research protocols for the use of human blood cells were approved by the Research Ethics Review Board of the Institut de recherches cliniques de Montréal (IRCM) in accordance with the declaration of Helsinki. Peripheral blood mononuclear cells (PBMCs) were recovered from buffy coats, following Ficoll-Paque density gradient separation (GE Healthcare, Little Chalfont, UK). Primary CD4+ T cells were isolated by negative selection using the human CD4+ T cell isolation kit from Miltenyi Biotech (Bergisch Gladbach, Germany), activated with 5 μg/mL phytohaemagglutinin-P for 48 h and cultured in the presence of 100 U/mL interleukin (IL)-2. Monocytes were isolated by adherence for 2 h in RPMI-1640 and then harvested directly in PBS containing 5 mM ethylenediaminetetraacetic acid (PBS-EDTA) following several gentle washes and scraping. Alternatively, monocytes were cultured 7 days in RPMI-1640 supplemented with 5% decomplemented human blood plasma to obtain MDMs. Monocyte and MDMs were regularly characterized by immuno-cytofluorometry and purity was determined at >90% as assessed by CD11c expression. As expected, following monocyte to macrophage differentiation, levels of CD14 were significantly down-regulated and CD16 up-regulated in MDMs. Macrophages were cultured in complete RPMI-1640 supplemented with 10% FBS following differentiation; in most cases, MDMs were transferred to 6-well plates for further analyses following treatment with Accutase (Affymetrix/eBioscience, Santa Clara, CA, USA) and gentle scraping.

Transfection of differentiated THP-1 cells or MDMs with Exiqon microRNA antagomirs was performed using Lipofectamine RNAimax (Invitrogen, Carlsbad, CA, USA). Briefly, a mix of 0.5 pmol of each antagomir and 8 µL of transfection reagent were each diluted into 150 µL of Opti-MEM (Invitrogen), mixed and added dropwise to 200,000 cells per well in a 6-well plate. Following 48 h of incubation, cells were harvested for quantitative real-time PCR (qRT-PCR) or flow cytometry analyses.

Unless otherwise indicated, MDMs were infected with HIV-1 at a multiplicity of infection (MOI) of 1 and THP-1 cells infected at a MOI of 5, based on the TZMbl assays. CD4+ T cells were maintained in RPMI-1640 supplemented with 10% FBS and 100 U/mL IL-2 and were infected at an MOI of 0.1.

### 2.6. RNA Extraction, Reverse-Transcription and qRT-PCR Analyses

Total cellular RNAs were extracted using RNeasy RNA extraction columns (Qiagen, Hilden, Germany) according to the manufacturer’s instructions and stored at −80 °C. For microRNAs, cDNAs were obtained by using either the NCode (Invitrogen) or High Specificity (Agilent Technologies, Santa Clara, CA, USA) microRNA First-Strand cDNA synthesis kits: briefly, 100–300 ng of total RNAs were poly-adenylated and an aliquot reversed transcribed using the SuperScript III reverse transcriptase. All other cDNAs were obtained using SuperScript II reverse transcriptase (Invitrogen) according to the manufacturer’s instructions. For qRT-PCR, cDNA and appropriate primers [26] were added to SybrGreen Select Master Mix (Applied Biosystems, Foster City, CA, USA) in 96 well plates and run on a ViiA96 thermocycler (ThermoFisher Scientific). For microRNA quantitation, primers were designed according to Balcells et al. [40] and were previously described [26]. Glyceraldehyde 3-phosphate dehydrogenase (*GAPDH*) gene expression was used as loading control and ΔΔ threshold cycle (ΔΔCT) variations calculated in all cases. Particular attention was noted on melt curve analyses to obtain optimal conditions.

### 2.7. Flow Cytometry

Undifferentiated THP-1 cells or monocytes were centrifuged, whereas differentiated THP-1 cells or MDMs were collected following a 15 min, 37 °C incubation in PBS-EDTA and gentle scraping. Harvested cells were then washed in PBS containing 2% FBS (FACS buffer) and blocked on ice 30 min in FACS buffer containing 2% goat serum, 2% rabbit serum and 500 µg/mL human IgG. Fluorochrome-labeled antibodies were added directly into the cells in the blocking solution, incubated 1 h on ice, washed twice in FACS buffer and fixed with 4% paraformaldehyde in PBS. Finally, cells were re-suspended in PBS-EDTA and analyzed on a BD Fortessa cytometer (San Jose, CA, USA) equipped with appropriate lasers. Detailed analyses were obtained using the FlowJo software package (version 9.3.2, Ashland, OR, USA).

### 2.8. SDS-PAGE and Western Immuno-Blotting Analyses

THP-1 cells were lysed in radioimmunoprecipitation buffer (RIPA) [33] containing EDTA and protease inhibitors (Roche, Basel, Switzerland) and 25 μg of lysate used for 10% sodium dodecyl sulfate polyacrylamide gel electrophoresis (SDS-PAGE). Following transfer onto nitrocellulose and soaking in blocking solution (5% milk in Tris buffered solution (TBS) with Tween 20), membranes were treated with rabbit anti-human CD4 (H370; Santa Cruz, Dallas, TX, USA) or rabbit anti-GAPDH (Biolegend), all diluted 1/1000. Following washes and addition of anti-rabbit horseradish peroxidase-conjugated antibodies (1/4000; Bio-Rad, Hercules, CA, USA), proteins were revealed by chemiluminescence.

### 2.9. Virus Encoding Luciferase Infection Assay

Vesicular stomatitis virus G glycoprotein (VSV-G) or HIV-ADA-Env pseudotyped NL4-3Env-Luc+Vpr+ (“NL4.3Luc”) viruses were used to infect cells that were previously treated with controls or a mix of miR-221 and miR-222 antagomirs, as described above. Cells were cultured for an additional 24 h and lysed in Cell Lysis Buffer (Promega, Madison, WI, USA). Luciferase activity in cell lysates was measured using the Dual-Glo Luciferase Assay System (Promega) on a GloMax luminometer (Promega).

### 2.10. Analysis of HIV-1 Replication Kinetics

Cells were infected either with NL4-3-ADA-IRES-GFP, its Vpr-negative version, or NL4-3-GFP. Cells and their supernatants were harvested at the indicated time points and viral production was determined by measuring HIV-1 p24 in the supernatants by enzyme-linked immuno-sorbent assay (HIV-1 p24 ELISA, XpressBio, Frederick, MD, USA) while viral spread was evaluated by assessing the frequency of GFP-positive cells by flow cytometry.

### 2.11. RNAseq of Primary Monocyte versus MDM MicroRNAs

Total RNA extracted from monocytes and corresponding MDMs (PBMCs from 2 blood donors) were used for microRNA sequencing with the Illumina TruSeq Small RNA system (Illumina Technologies, San Diego, CA, USA) at the IRCM Molecular Biology and Functional Genomics Core Facility. Specific tagging was used to identify RNA from each blood donor and both cell types. The resulting library was sequenced at the Core Facility using 50 bp paired-end (PE50) sequencing on a HiSeq 2000 sequencer (Illumina Technologies). Sequences where then processed at the IRCM Bioinformatics Core Facility. Adaptor sequences and poor-quality bases were trimmed with Cutadapt and analysed RNAs set at 17–35 base pairs. Alignment and quantification of individual microRNAs were performed using miRDeep2 with the miRbase.org (v.21, University of Manchester, Manchester, UK) database. Differential expression of microRNAs was assessed using adjusted *p* values computed using DESeq2. Heat maps were generated using R statistical software. The accession number for the microRNAseq data reported in this study is GEO: GSE107160.

### 2.12. Statistics

Statistical analyses were performed using either Student’s *t* or Mann-Whitney U tests, as indicated, using GraphPad Prism v7 (GraphPad Software, LaJolla, CA, USA). The following symbols are used throughout the manuscript: * *p* < 0.05; ** *p* < 0.01; *** *p* < 0.001 and **** *p* < 0.0001.

## 3. Results

### 3.1. MiR-221 and -222 Are Up-Regulated in Monocyte-To-Macrophage Differentiation

**Figure 1:**
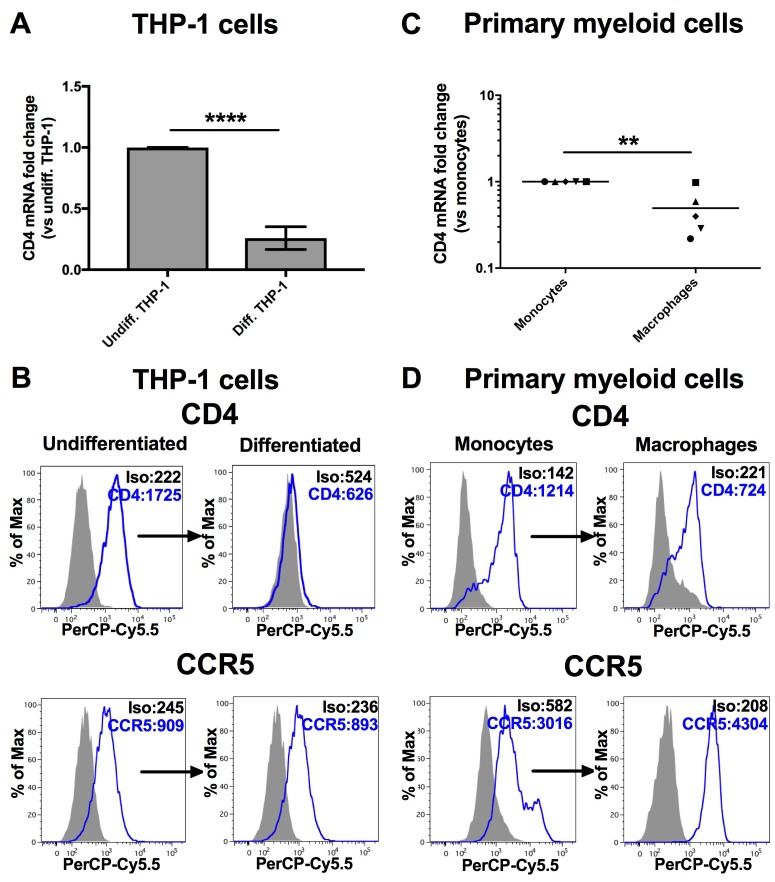
CD4 expression is reduced following differentiation of monocytes into macrophages. ** *p* < 0.01 and **** *p* < 0.0001. The levels of CD4 mRNA (**A**: *n* = 12; means ± SD are shown and compared using Student’s *t* test), CD4 or CCR5 at the cell surface (**B**: representative graphs are shown, *n* = 4) were compared in undifferentiated THP-1 cells or differentiated THP-1 cells following PMA treatment. Likewise, CD4 mRNA (**C**: *n* = 5 blood donors; bars represent mean values which are compared using Mann-Whitney’s U test) or surface expression of CD4 or CCR5 (**D**: representative graphs are shown of *n* = 4) were also measured in human primary monocytes and their corresponding derived macrophages (MDMs). Mean geometric fluorescence intensities (MFIs) are shown in the histograms.

Monocytes undergo important functional and morphological changes upon their differentiation into macrophages. For example, the expression of CD4 is significantly reduced in human macrophages as compared to monocytes [7,9], despite the fact that macrophages are more susceptible to HIV infection than monocytes [17,18]. We set out to further investigate factors that regulate CD4 mRNA expression in monocyte-to-macrophage differentiation. In order to confirm the modulation of CD4, we first compared the levels of both CD4 mRNA and surface expression in monocytes and derived macrophages of either human primary myeloid cells or the established THP-1 human monocytic cell line that can be differentiated into adherent macrophage-like cells following PMA treatment [41]. As shown in Figure 1, expression of CD4 mRNA is lowered by 4-fold in PMA-differentiated THP-1 cells as compared to their undifferentiated counterparts (*p* < 0.0001, Student’s *t* test, Figure 1A); strikingly, CD4 at the cell surface is reduced to barely detectable levels (*p* = 0.01, Student’s *t* test, Figure 1B). In primary myeloid cells, we also observed both a significant mean reduction of CD4 mRNA (*p* = 0.008, Mann-Whitney’s U test) (Figure 1C) and cell surface expression (*p* = 0.03, Mann-Whitney’s U test) in monocyte-derived macrophages (Figure 1D).

**Figure 2:**
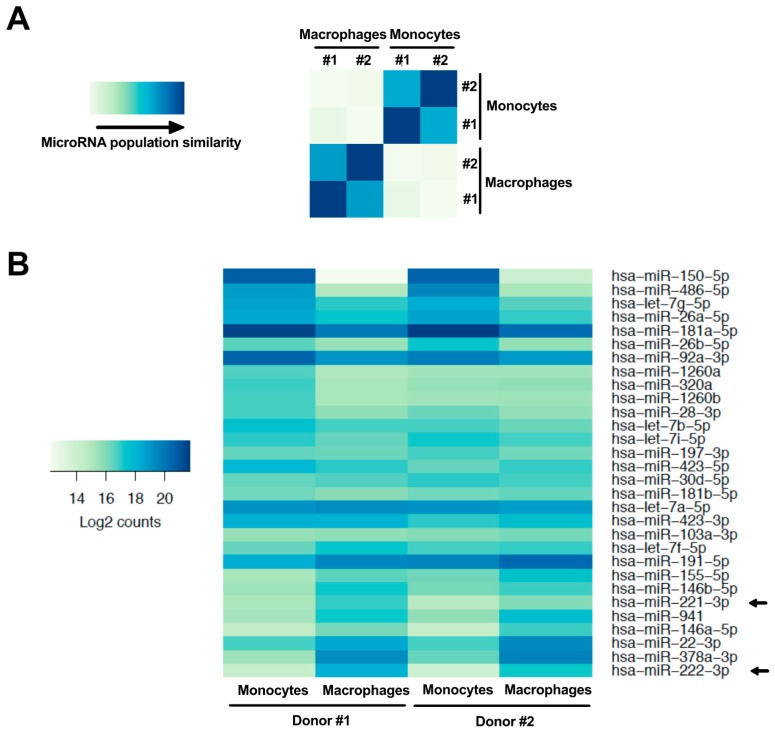
MicroRNA expression is modulated following monocyte differentiation into macrophages. (**A**) Heat map comparing the relative expression of monocyte and macrophage microRNAs in cells derived from the PBMCs of 2 different donors (#1 and #2), as obtained by microRNAseq (the blue gradient represents the level of similarity between two populations; the darkest blue representing equivalent populations). (**B**) Heat map showing the relative expression (darkest blue representing strongest expression; log2[macrophage mean read counts/monocyte mean read counts]) in both cell types of the 30 most highly expressed microRNAs in monocytes or macrophages. Arrows identify miR-221 and miR-222.

MicroRNAs are important regulators of mRNA expression and are modulated in monocyte-to-macrophage differentiation [21,24]. Given that CD4 mRNA is significantly reduced in this differentiation process, we compared, using RNAseq, the microRNA profile of primary human monocytes and derived macrophages of 2 blood donors in order to identify microRNAs that could regulate CD4 mRNA during the differentiation. As shown in the Figure 2A heat map, the monocyte microRNA populations from both blood donors undergo important changes following differentiation into MDMs. Of the 1394 microRNAs analyzed, 341 were at least expressed 2-fold more in monocytes as compared to macrophages and 279 were up-regulated at least 2-fold following differentiation (Table S1; the fold changes are represented in log2[macrophage expression/monocyte expression]). When we compared the profile of the 30 highest expressed myeloid microRNAs (i.e. in either monocytes or macrophages, according to the base mean values of Table S1), several microRNAs displayed enhanced or reduced expression in MDMs when compared to monocytes (Figure 2B). Indeed, half of these microRNAs showed a 2-fold change in expression following monocyte-to-macrophage differentiation. A complete list of the microRNA expression profile is shown in Table S1.

**Figure 3:**
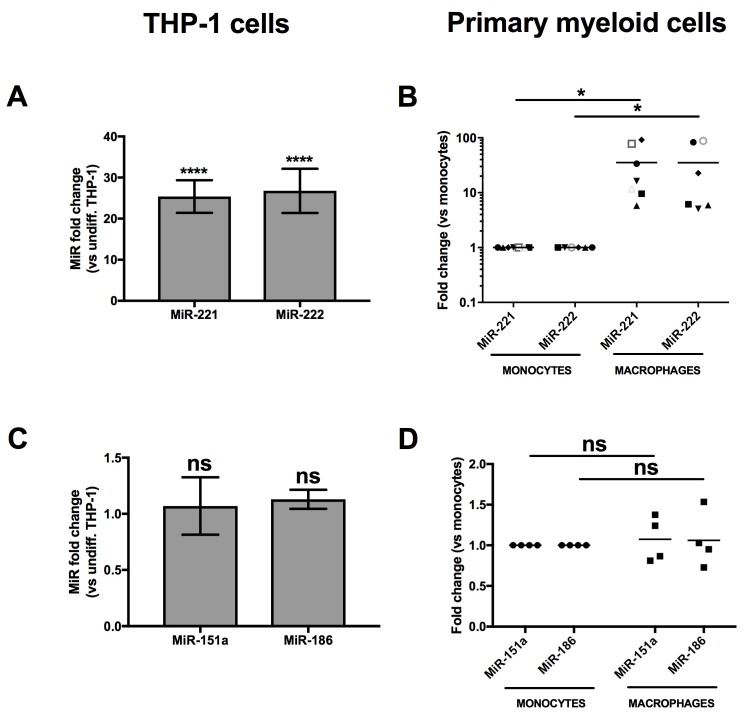
MiR-221 and miR-222 expression is enhanced in THP-1 cells or MDMs following differentiation. * *p* < 0.05 and **** *p* < 0.0001. The expression of miR-221 or miR-222 was compared in THP-1 cells prior or after differentiation (**A**: *n* = 11; means ± SD are shown and compared using Student’s *t* test), or monocytes and corresponding macrophages from at least 6 blood donors (**B**: *n* = 6 donors represented by different symbols; bars represent mean values which are compared using Mann-Whitney’s U test), respectively, by qRT-PCR. MiR-151a and miR-186, which expression did not significantly change in the RNAseq (Figure 2), were also analyzed in THP-1 (**C**: *n* = 3) or primary cells (**D**: *n* = 4 blood donors) as validation controls.

Of the highly expressed microRNAs shown in Figure 2B, the most enhanced following monocyte-to-macrophage differentiation was miR-222 (*p* = 3.7 × 10^−10^, log2 = 3.5), which we have recently shown to be a regulator of CD4 mRNA in macrophages [26]. This microRNA is derived from a pri-microRNA precursor from which miR-221 is also processed. Accordingly, miR-221 (*p* = 8.4 × 10^−3^, log2 = 1.5) was also enhanced in MDMs (Figure 2B). Both miR-221 and miR-222 are paralogues that recognize the identical seed sequence in the 3′UTR of CD4 mRNAs [25,26]. In order to validate our RNAseq data, we measured both miR-221 and miR-222 expression by qRT-PCR in THP-1 or primary human monocytes and compared their expression to that in corresponding derived macrophages (Figure 3). Both miR-221 and miR-222 were highly and significantly enhanced (both ~25-fold) in THP-1 cells following PMA-induced differentiation (Figure 3A). Moreover, such stimulation was also observed in primary MDMs (Figure 3B), as compared to monocytes; both microRNAs were enhanced more than 30-fold in macrophages following 7 days of differentiation. On the other hand, levels of either miR-151a or miR-186, which did not change in differentiation according to RNAseq, were also found to be similar by qRT-PCR in both THP-1 or primary myeloid cells following differentiation (Figure 3C,D). These results suggest that CD4 receptor down-regulation occurring during the differentiation of monocyte to macrophages might be linked to the increased expression of miR-221 and miR-222.

### 3.2. Establishment of a THP-1 Cell Line Expressing CD4 that Is Not Regulated by MiR-221 or MiR-222

**Figure 4:**
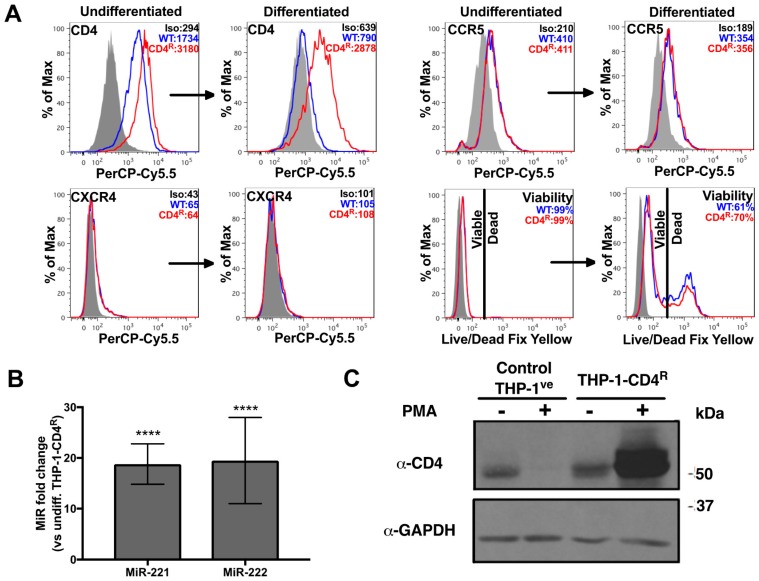
THP-1-CD4^R^ cells express CD4 independently of miR-221 and miR-222 modulation. **** *p* < 0.0001. (**A**) Either undifferentiated or PMA-differentiated THP-1-CD4^R^ cells (or the control THP-1^ve^ cells, WT) were analyzed for their viability and the surface levels of CD4, CXCR4 or CCR5 by cytofluorometry. Representative graphs are shown. Mean geometric fluorescence intensities (MFIs) or viability values are shown in the histograms. (**B**) The expression of miR-221 or miR-222 was compared in THP-1-CD4^R^ cells prior or after differentiation (*n* = 12; means ± SD are shown and compared using Student’s *t* test). (**C**) Expression of CD4 was measured by western blot in either control THP-1^ve^ or THP-1-CD4^R^ cells, prior or following PMA-induced differentiation. A representative blot is shown.

In order to establish a direct link between CD4 mRNA down-regulation and miR-221/miR-222 up-regulation during monocyte to macrophage differentiation, we took advantage of the THP-1 model system (Figure 1B) and established a THP-1 cell line expressing a *CD4* transgene resistant to miR-221/miR-222 modulation. THP-1 cells were thus transduced with a retroviral vector containing the *CD4* gene in which the complete 3′UTR, including the miR-221/miR-222 target sequence, is deleted (hereafter named *CD4*(Δ3′UTR)) [26]. The hygromycin-resistant, THP-1-CD4^R^ cell line obtained was then examined for CD4, CXCR4 and CCR5 expression and compared to THP-1 cells transduced with control vectors (THP-1^ve^). Both undifferentiated THP-1-CD4^R^ and THP-1^ve^ cells expressed very low, comparable levels of CXCR4 but displayed moderate levels of CCR5 (Figure 4A). Thus, both cell lines reacted similarly to differentiation as to their CXCR4 or CCR5 phenotypes. In contrast, although surface CD4 expression levels were higher in undifferentiated THP-1-CD4^R^ as compared to THP-1^ve^ controls, differentiated THP-1-CD4^R^ cells maintained a strong surface CD4 expression as compared to the reduced, near background levels of CD4 in differentiated THP-1^ve^ cells (Figure 4A), despite the up-regulation of miR-221 and miR-222 (Figure 4B). The total expression of CD4 was also analyzed by western blotting in these cells in differentiated and undifferentiated conditions (Figure 4C); we observed only a limited increase in total CD4 expression in undifferentiated THP-1-CD4^R^ as compared to undifferentiated control THP-1^ve^ cells, which is likely due to the miR-221/miR-222 refractory *CD4*(Δ3′UTR) transgene being under the control of a strong cytomegalovirus immediate early (CMV-IE) promoter. Indeed, this promoter is poorly active in monocytes but very active in macrophages [42]. Interestingly, in contrast to THP-1^ve^ control cells, total CD4 expression levels were drastically increased in THP-1-CD4^R^ cells upon differentiation (Figure 4C). This enhancement of total CD4 expression levels in differentiated THP-1-CD4^R^ did not result into a proportional increase in surface CD4 levels (Figure 4A); indeed, expression levels of surface CD4 remained comparable to those detected in undifferentiated THP-1-CD4^R^ cells (Figure 4A), a condition likely due to endoplasmic reticulum obstruction and/or saturation of transport pathways to the plasma membrane. Finally, both undifferentiated cell lines exhibited excellent viability, although following their differentiation, some cell death (30–40%) was evident (Figure 4A), likely due to the strong PMA-induced stimulation.

**Figure 5:**
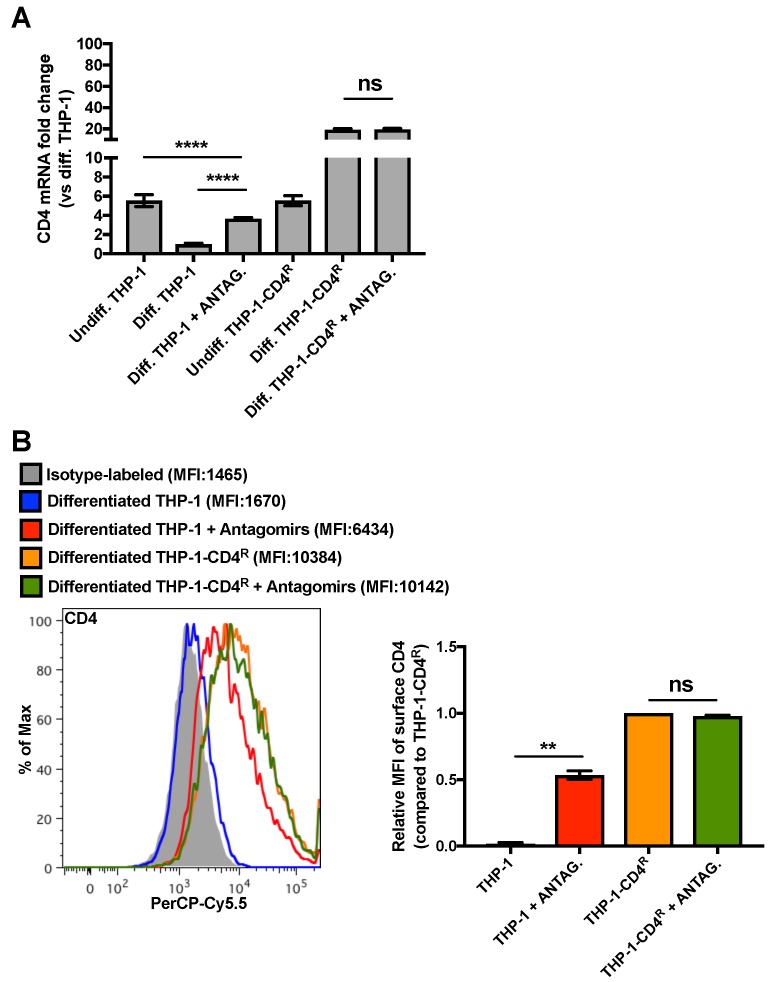
MiR-221/miR-222 antagomirs restore CD4 expression in differentiated THP-1 cells to that of levels in undifferentiated THP-1 cells. ** *p* < 0.01 and **** *p* < 0.0001. (**A**) Control THP-1 or THP-1-CD4^R^ cells were PMA-differentiated and transfected with a mix of miR-221/miR-222 antagomirs. CD4 mRNA levels were then measured by qRT-PCR (*n* = 6; means ± SD are shown and compared using Student’s *t* test). (**B**) The levels of surface CD4 of the previously described cells were assessed by cytofluorometry; a graph of a representative experiment is shown (left panel; geometric MFIs are shown in parentheses) and a bar graph of the relative geometric MFIs (*n* = 2; means ± SD are shown and compared using Student’s *t* test) is provided in the right panel.

Since the total 3′UTR is deleted in *CD4*(Δ3′UTR) mRNA and this region may contain other regulatory microRNA target sites than that of miR-221/miR-222, we transfected a mix of miR-221- and miR-222-specific antagomirs in THP-1 or THP-1-CD4^R^ differentiated cells in order to specifically inhibit these microRNAs and measure the resulting impact on CD4 mRNA and surface expression. As shown in Figure 5, miR-221/miR-222 antagomir transfection has no apparent effect on CD4 expression in differentiated THP-1-CD4^R^ cells. However, antagomir treatment in control differentiated THP-1 cells significantly enhanced CD4 mRNA and cell surface expression (Figure 5), although CD4 mRNA levels did not reach that of undifferentiated THP-1s. Taken together, these results indicate that the CD4 receptor down-regulation observed upon differentiation of monocyte into macrophages is likely the direct result of miR-221- and miR-222-mediated targeting of CD4 mRNAs.

### 3.3. Differentiated THP-1-CD4^R^ Cells Are Efficiently Infected by HIV-1

**Figure 6:**
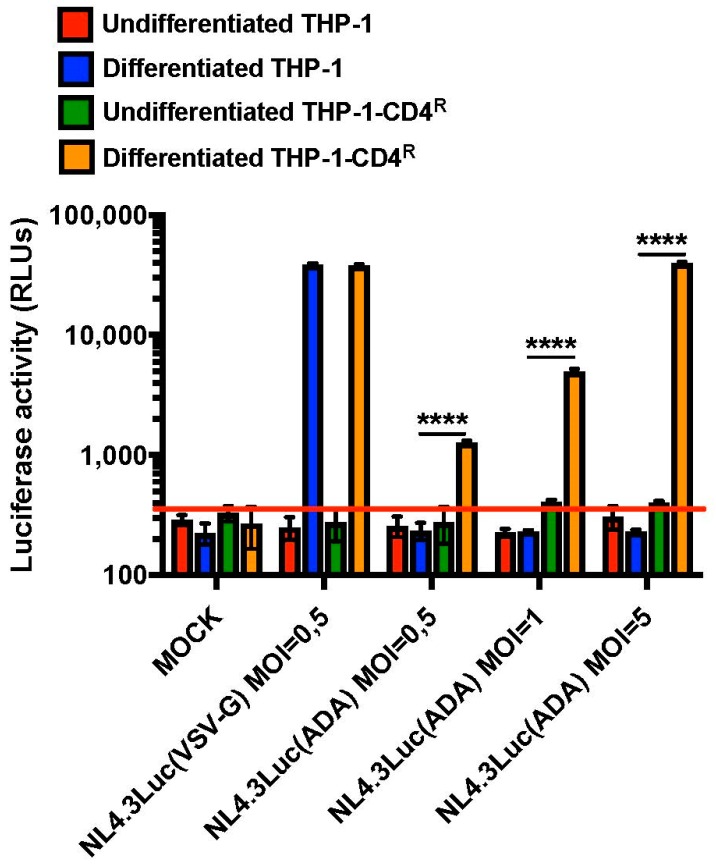
Differentiated THP-1-CD4^R^ cells are susceptible to CD4-dependent, HIV-1 infection. **** *p* < 0.0001. Control THP-1 or THP-1-CD4^R^ cells were either PMA-differentiated or not and infected with either VSV-G (MOI = 0.5) or HIV-ADA-Env (MOIs of either 0.5, 1 or 5)-pseudotyped Luciferase-encoding NL4-3 Env-negative Luc viruses. Luciferase activity was then measured in the cell lysates and expressed as relative light units (RLUs) (*n* = 3; means ± SD are shown and compared using Student’s *t* test). The red line indicates background level. ‘MOCK’ corresponds to uninfected cells.

Having shown that the levels of CD4, the principal cellular receptor of HIV-1, could be maintained upon differentiation of the THP-1-CD4^R^ cell line, we analyzed the susceptibility of these cells to HIV-1 infection and importantly examined whether HIV-1 functions that are phenotypically assessable only in primary macrophages could be studied in the THP-1-CD4^R^ cell line. Either undifferentiated THP-1 or THP-1-CD4^R^ cells, or their derived PMA-differentiated counterparts, were infected with different HIV-1 Luciferase-reporter viruses capable of a single round of replication as described in Figure 6. Viruses coated with the vesicular stomatitis virus (VSV) G glycoprotein, which entry is CD4-independent, efficiently infected either differentiated THP-1 or THP-1-CD4^R^ cells but not their undifferentiated monocytic counterparts, as monocytes are reported to be non-permissive to HIV infection in part because of post-entry blocks [17,18] (Figure 6). However, viruses pseudotyped with the CCR5-tropic HIV-1 ADA-Env were only able to efficiently infect differentiated THP-1-CD4^R^ cells, although a 10-fold increase in infectious virus input was necessary to reach levels of infection comparable to VSV-G-pseudotyped HIV-1. Nevertheless, these data show that differentiated THP-1-CD4^R^ cells, unlike their parental THP-1 cells, can be efficiently infected with HIV-1 in a CD4-dependent manner, without the additional need of pan-tropic glycoproteins, such as VSV-G.

**Figure 7:**
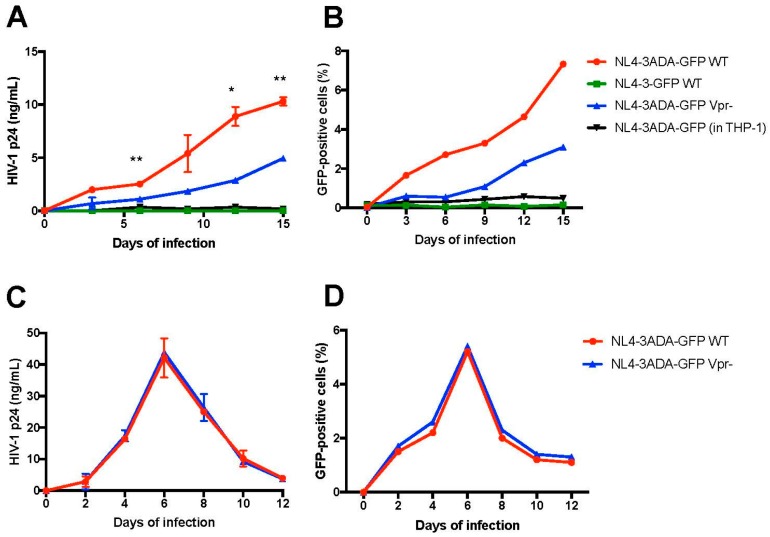
Differentiated THP-1-CD4^R^ cells sustain active HIV-1 replication and spread. * *p* < 0.05 and ** *p* < 0.01. Differentiated THP-1-CD4^R^ cells were infected (MOI = 5) with either WT (Vpr+) or Vpr-negative (Vpr−) NL4-3ADA-GFP virus (CCR5-tropic) virus, or NL4-3-GFP virus (CXCR4-tropic) and the levels of HIV-1 p24 in the supernatant (**A**) and GFP-positive cells (**B**) measured over time. Differentiated THP-1 cells were infected with WT (Vpr+) virus as controls. Alternatively, CD4+ T cells were infected with the same viral stocks (MOI = 0.1); HIV-1 p24 in the supernatant (**C**) and GFP expression (**D**) were assessed over time. For HIV-1 p24, the mean p24 levels ± SD (*n* = 2) are shown and compared using Student’s *t* test at selected time points. WT and Vpr− viruses showed no significant difference in kinetics in CD4+ T cells.

We next asked if differentiated THP-1-CD4^R^ cells are permissive for productive HIV-1 infection and as such able to sustain virus spread in culture. THP-1-CD4^R^ differentiated cells were infected with a GFP-encoding, fully replicative CCR5-tropic HIV-1 (NL4-3ADA-GFP) (MOI = 5) and the levels of GFP-positive cells and HIV-1 p24 in the culture supernatants measured over time by flow cytometry and ELISA, respectively. As shown in Figure 7, infected cells produced newly infectious HIV-1 as demonstrated by an increase of HIV-1 p24 in the supernatants as well as by an augmentation of the number of GFP-positive infected cells over time. This sharply contrasted with undifferentiated THP-1-CD4^R^ cells, or either undifferentiated or differentiated parental THP-1 cells, which did not sustain any detectable HIV-1 infection (THP-1 differentiated cells infected with CCR5-tropic HIV-1 are shown as controls in Figure 7). No detectable infection in undifferentiated or differentiated THP-1-CD4^R^ cells was obtained using a control, CXCR4-tropic virus (NL4-3-GFP, Figure 7), consistent with the very low levels of CXCR4 expression detected in these cells (Figure 4A).

To take fully advantage of this feature of the THP-1-CD4^R^ line, we tested in the HIV-1 infection assay a Vpr deficient version of the CCR5-tropic virus. Indeed, HIV-1 requires Vpr for optimal viral replication and spread in primary macrophages [32,43,44] but the precise mechanisms underlying how Vpr facilitates infection in macrophages are not well understood. As shown in Figure 7, a significant decrease in virus replication, as measured by HIV-1 p24 (around 2–3 fold), was detected in supernatants from Vpr-negative virus infected cells, as compared to those infected with the wild-type HIV-1. As expected, Vpr-defective viruses displayed also a diminished rate of spread in the culture (Figure 7B). However, as previously reported [45], WT and Vpr-defective viruses showed similar replication in dividing T cells (Figure 7C,D). Thus, the Vpr-negative HIV-1 showed a delay in replication kinetics in differentiated THP-1-CD4^R^ cells, similarly to what has been reported in primary macrophages.

## 4. Discussion

There are several advantages in establishing a practical monocytic model cell line for the study of HIV-1 infection in macrophages, given the variability and processing challenges of primary monocyte-derived macrophages. In this context, Cassol et al. [46] compared the use of several (MonoMac1, THP-1, U937 and HL-60) monocyte/macrophage lines as HIV-1 infection models. Of these, only the MonoMac1 and THP-1 lines seem to keep a stronger morphological resemblance to macrophages once differentiated. However, a major shortcoming of THP-1 cells is their high expression levels of several HIV-1 infection restriction factors, such as APOBEC and SAMHD1 [47,48,49], although in the case of SAMHD1, the presence of restriction inactive (phosphorylated) or active (unphosphorylated) forms of the restriction factor are reported to vary according to the differentiation status of THP-1 [50]. In addition to this, the use of phorbol diester PMA in order to induce THP-1 differentiation, results in the activation of several cellular pathways through NF-κB [51] that may give rise to adverse, undesired effects. Differentiated THP-1 cells possess very low levels of CD4 at their surface (Figure 1), which necessitates pseudotyping incoming HIV-1 with other viral glycoproteins (such as VSV-G) to obtain efficient infection, though CD4-independent, viral entry [52]. Although HIV-1 spread has been reported in differentiated THP-1 cells [41], it is strongly delayed. This would be consistent with low CD4 expression being a major hurdle for efficient HIV-1 infection in differentiated THP-1s [41]. We here report and characterize a new, THP-1-derived line, THP-1-CD4^R^, in which CD4 expression is released from miR-221/miR-222 regulatory processes, resulting in high levels of CD4 expression following PMA-induced differentiation, enabling CD4-dependent infection by HIV to efficiently take place.

Monocytes express significantly higher levels of CD4 than macrophages [7,9] and the precise mechanisms governing the reduction of CD4 expression levels during monocyte to macrophage differentiation remain poorly understood. We performed an analysis of the microRNA expression profile of primary human monocytes and their derived macrophages to gain further insight on how CD4 modulation is taking place. Interestingly, we found that miR-28-3p (*p* = 5 × 10^−2^, log2 = −0.9), miR-150-5p (*p* = 3.2 × 10^−21^, log2 = −6.5), miR-223-3p (*p* = 9 × 10^−2^, log2 = −1.34) and miR-382-5p (*p* = 1 × 10^−4^, log2 = −4.26), which have been reported to target HIV-1 RNAs [21,53,54], were enhanced in monocytes (Figure 2 and Table S1), providing examples of microRNAs participating in the reduced permissiveness of monocytes to HIV-1 infection. Among the microRNAs enhanced in macrophages were miR-155-5p (*p* = 7.4 × 10^−2^, log2 = 1.1), a microRNA that has been shown to down-regulate the HIV-dependency factors, LEDGF/p75, ADAM10 and NUP153 in TLR3-activated macrophages [55], miR-146a (*p* = 7 × 10^−5^, log2 = 2), which promotes HIV-1 infection in microglial cells [56], miR-221 (*p* = 8.4 × 10^−3^, log2 = 1.5) and miR-222 (*p* = 3.7 × 10^−10^, log2 = 3.5) [57]. An increase of miR-221 and miR-222 was also observed during differentiation of monocytic THP-1 cells, a finding consistent with previously reported data [58] (Figure 3).

We have recently shown that miR-221 and miR-222 are regulators of CD4 mRNA in macrophages [26]. The CD4 reduction in the differentiation process also coincides with the up-regulation of these microRNAs in monocyte-to-macrophage differentiation. The negative regulation of CD4 expression during differentiation may be linked to its reported function in this process. Indeed, it was reported that CD4 ligation to MHC-II induces differentiation of blood monocytes into macrophages [59], possibly leading to its subsequent reduced expression through miR-221 and miR-222. In the case of HIV-1 infection, the levels of CD4 in macrophages appear to have a direct impact on the susceptibility of these cells to HIV-1 infection as well as on the properties that CCR5-tropic Env need to have to mediate viral entry in macrophages [60]. MiR-221 and miR-222-mediated CD4 down-regulation may indeed contribute to restricting viral entry in macrophages to CCR5-tropic viruses encoding envelope with high affinity for CD4. The susceptibility of other myeloid cells to HIV-1 infection appears modulated by surface CD4 expression levels. Recent evidence suggest that monocyte-derived dendritic cells (MDCC) are poorly infected by various HIV-1 and HIV-2 strains in part because of low CD4 levels [61]. Whether this property of MDCCs results from enhanced expression of miR-221 and miR-222 will be interesting to investigate.

While miR-221 and miR-222 are up-regulated comparably in macrophages and in differentiated THP-1 cells, their impact on CD4 mRNAs and surface proteins levels was found to be significantly different (Figure 1 and Figure 3). Interestingly, quantitative analysis revealed that miR-221 and miR-222 are expressed in MDMs in the range of 3907 ± 667 and 3177 ± 555 copies per cell, respectively, whereas their absolute levels are in the range of 15,116 ± 2263 and 26,670 ± 3496 copies per cell, respectively in undifferentiated THP-1 cells [26]. The fact that more miR-222 is found in THP-1 cells, as compared to miR-221, may be due to different levels of post-transcriptional pri-microRNA processing events in the transformed THP-1 cells which are not present in primary monocytes/macrophages. Moreover, these data also suggest that the absolute levels of these microRNAs in macrophages and differentiated THP-1 cells is likely to differ by ~2 orders of magnitude, a condition that is likely to impact differently CD4 expression levels in these two cellular environments. PMA used to induce THP-1 differentiation has been reported to enhance NF-κB-driven genes [51], such as the one encoding miR-221/miR-222. To further show that CD4 is modulated by these microRNAs in differentiation, we established a THP-1-derived cell line (THP-1-CD4^R^) expressing CD4 mRNA deleted in the 3′UTR, which contains the sites recognized by miR-221/miR-222. In these cells, expression of the exogenous *CD4*(Δ3′UTR) is driven by a CMV promoter, which is only efficient in differentiated THP-1-CD4^R^ cells, where endogenous CD4 is down-regulated. Although the levels of surface CD4 reach high levels in both undifferentiated and differentiated THP-1-CD4^R^ cells (Figure 4), it is noteworthy that the total expression level of exogenous *CD4*(Δ3′UTR) in differentiated THP-1-CD4^R^ cells is much greater than endogenous CD4 in their undifferentiated counterparts (Figure 4C), suggesting that much exogenous CD4 is trapped in the endoplasmic reticulum/Golgi secretory pathway. However, the fact that CD4 expression (Figure 4) and mRNA (Figure 5) in the THP-1-CD4^R^ cells remained high following differentiation, in contrast to THP-1 control cells, suggests that *CD4*(Δ3′UTR) expression is not modulated by miR-221/miR-222. This was also supported by the fact that miR-221/miR-222 antagomirs in PMA-differentiated THP-1-CD4^R^ cells had no significant effect on CD4 mRNA or surface expression (Figure 5), whereas transfection of these antagomirs in differentiated THP-1 cells significantly enhanced CD4. It is noteworthy that although surface levels of CD4 were greatly enhanced in antagomir-transfected differentiated THP-1 cells, antagomir transfection in these cells did not elevate CD4 mRNA to levels found in their undifferentiated counterparts, possibly due to transfection efficiency.

Undifferentiated THP-1 cells have been reported to be susceptible to HIV-1 infection [62,63] in part because SAMHD1 is in a phosphorylated restriction inactive form [50]. In our experimental conditions, we were not able to detect Env-dependent nor Env-independent viral infection in undifferentiated THP-1 and THP-1-CD4^R^ cells as shown in Figure 6. Whether this is due to the virus genetic background (*Nef*-defective) used in our experiments or properties of the THP-1 clones that we selected remains currently unclear. In contrast, differentiated THP-1-CD4^R^ cells could be infected in a single round assay by HIV-1 in a CD4-dependent manner, although 10-fold more HIV-Env-pseudotyped reporter virus was needed to reach levels comparable to its VSV-G-pseudotyped counterpart (Figure 6). Thus, a possible additional limiting factor for CD4-mediated entry may be the low levels of CCR5 co-receptors detected in THP-1 cells (Figure 4). Nevertheless, THP-1-CD4^R^ cells were able to sustain HIV-1 replication and spread of infection in culture (Figure 7) comparable to levels in primary human MDMs [26], suggesting that post-entry blocks are comparable in both instances, although we cannot exclude the possibility that the high levels of CD4 presumably trapped intracellularly in the THP-1-CD4^R^ cells might impair to some extent viral spread by altering Env incorporation into progeny virions. The fact that THP-1-CD4^R^ cells are able to sustain HIV-1 replication and spread opens several possibilities as to their use to study HIV-1-macrophage interactions. For instance, THP-1-CD4^R^ cells are more easily amenable to siRNA and CRISPR studies. Moreover, *Vpr*-negative viruses show delayed replication kinetics in THP-1-CD4^R^ cells, reinforcing their potential as a model for studying HIV-1 infection of macrophages, without the complications involved in deriving MDMs and manipulating blood-derived primary cells. Indeed, macrophages require Vpr for optimal spread of infection [32,43] and THP-1-CD4^R^ cells could be used to investigate how Vpr facilitates infection, given that this phenotype is only detected in spreading viral infections [44,64]. Taken together, these findings show that THP-1 cells are a valuable model for the study of HIV-1 infection in macrophages and further supports the use of the THP-1-derived, THP-1-CD4^R^ cell line in this context.

## 5. Conclusions

Besides modulating CD4 expression in activated macrophages and bystander macrophages during HIV-1 infection [26], miR-221 and miR-222 participate in the phenotypic changes of monocyte/macrophage differentiation. By acting on CD4, these microRNAs control the susceptibility of differentiated THP-1 cells to HIV-1 infection. Stable release of CD4 from miR-221/miR-222 modulation in THP-1 cells, such as in the THP-1-CD4^R^ cell line, results in efficient and productive HIV-1 infection. Thus, miR-221/miR-222 act as modulators of CD4 mRNA expression throughout the myeloid cell lineage in differentiation-activation processes, in addition to modulating macrophage susceptibility to HIV-1 infection.

## Figures and Tables

**Table 1:** Primers used in this study ^1^.

Primer Name	Sequence	Use
CD4 F-seq	5′GCAGAACCAGAAGAAGGTG3′	CD4 forward sequencing
CD4 F	5′ATGAACCGGGGAGTCC3′	1st, 2nd, 3rd CD4 PCRs forward primer
CD4 R	5′AATGGGGCTACATGTCTTC3′	1st CD4 PCR reverse primer
Linker 6GLY R	5′TCCTCCTCCACCACCACCAATGGGGCTACATG3′	2nd CD4 PCR reverse primer
Linker V5 R	5′CGTAGAATCGAGACCGAGGAGAGGGTTAGGGATAGGCTTACCTCCTCCTCCACCA3′	3rd CD4 PCR reverse primer
BamHI F	5′CATCTGGATCCGCCACCATGAACCGGGGAG3′	4th CD4 PCR forward primer
EcoRI R	5′CACATGAATTCTTACGTAGAATCGAGAC3′	4th CD4 PCR reverse primer

^1^ Additional primers used are described in Lodge et al. [26].

## Supplementary Materials

The following are available online at www.mdpi.com/1999-4915/10/1/13/s1; Table S1: RNAseq showing differential expression of microRNAs in monocytes and macrophages. The expression profile of 1394 microRNAs obtained by RNAseq of monocytes and their corresponding derived macrophages (*n* = 2 blood donors) are shown. The base mean is the average expression of the microRNA in both monocytes and macrophages (combined). The log2 fold change corresponds to the log2[macrophage mean/monocyte mean], thus a positive log2 fold change corresponds to an up regulation in macrophages and a negative log2 fold change to that of a down regulation in macrophages following monocyte differentiation. Standard deviation of the log2 fold change is in column E. The statistical probability (*p* value) is derived from the log2 fold change and microRNAs are listed in order of log2 fold change.
